# A Quantitative Review of Brain Activation Maps for Mentalizing, Empathy, and Social Interactions: Specifying Commonalities and Differences

**DOI:** 10.3390/bs15070934

**Published:** 2025-07-10

**Authors:** Bela Kranewitter, Matthias Schurz

**Affiliations:** 1 Department of Psychology, University of Innsbruck, 6020 Innsbruck, Austria; bela.kranewitter@student.uibk.ac.at; 2 Department of Psychology and Digital Science Center, University of Innsbruck, 6020 Innsbruck, Austria

**Keywords:** social cognition, social interaction, theory of mind, meta-analysis

## Abstract

Humans are inherently social beings, and the quality of their interactions is essential for maintaining physical and mental health. Effective social interaction involves understanding not just people’s visible behavior but also the underlying factors like thoughts and emotions. This review investigates the convergence and divergence of meta-analytic brain activation for mentalizing, empathy, and social interaction engagement. To achieve this, we re-analyzed data from our prior meta-analysis on mentalizing and empathy using the same methodology as an existing meta-analysis on social interaction engagement. The comparison of brain activation maps focused on the question of whether the co-activation of cognitive and affective brain systems is an overarching characteristic of intermediate mentalizing/empathy tasks and social interaction engagement. Our findings support the general assumption that social interaction engagement co-recruits cognitive and affective brain systems also implicated in mentalizing and empathy. However, we found little direct overlap of brain activation for intermediate mentalizing/empathy tasks and social interaction engagement. Finally, applying a network neuroscience perspective, we suggest that social interaction engagement, affective/empathy, and intermediate mentalizing/empathy tasks are collectively characterized by co-recruitment of the default mode network and control networks.

## 1. Introduction

Human life unfolds within a complex network of relationships. Numerous studies have indicated that both the quality and the amount of social interactions play an important role in maintaining physical (Holt-Lunstad et al., 2010; Kim et al., 2016; Pressman et al., 2005) and mental well-being (Boss et al., 2015; Kurina et al., 2011; Kuiper et al., 2015). To engage successfully in social interactions, it is essential to keep track not just of the observable actions of other people, but also the underlying factors driving them, such as their thoughts and feelings.

This review article focuses on studying how the neural building blocks of mentalizing and empathy—two central components of social cognition—are involved in social interaction engagement. We note that we do not address other component processes implicated in social cognition, such as the action observation system (Hardwick et al., 2018; Rizzolatti & Craighero, 2004), which is relevant for understanding action goals and the intentions behind movements. While this and other components of social cognition are beyond the scope of our work, they have been discussed in other and more comprehensive review articles (e.g., Adolphs, 2009; Happé et al., 2017; Lieberman, 2007; Van Overwalle, 2009).

### 1.1. Mentalizing and Empathy

The term mentalizing describes “the ability to attribute mental states (e.g., knowledge, intentions, emotions, perception) to self and others”, whereas empathy can be defined as “the ability to experience others’ affective states, while maintaining the distinction from one’s own affective states” (Quesque et al., 2024; see also Eikelboom et al., 2025). Over the past few decades, mentalizing and empathy have been intensely studied, and behavioral and neuroimaging research has generated a variety of different operationalizations of the two concepts. This heterogeneity has been increasingly identified as an obstacle for understanding the component processes of mentalizing and empathy (Happé et al., 2017; Quesque & Rossetti, 2020; Schaafsma et al., 2015; Bloom, 2017; Zaki, 2017). Moreover, some previous theoretical models of mentalizing and empathy have also contained substantial overlap between concepts, such as affective mentalizing (e.g., Kalbe et al., 2010; Schlaffke et al., 2015; Sebastian et al., 2012; Shamay-Tsoory & Aharon-Peretz, 2007) and cognitive empathy (e.g., Hooker et al., 2010; Walter, 2012).

In order to address the heterogeneous operationalizations and processes involved in mentalizing and empathy, we have previously carried out a meta-analysis (Schurz et al., 2021), sorting the implicated tasks and stimuli based on a clustering of brain activation patterns. That is, we first sorted neuroimaging studies on the two topics based on stimuli and tasks, second computed individual meta-analytic brain activation maps per task type, and third applied hierarchical agglomerative clustering to identify common patterns of brain activation—and therefore organized operationalizations of mentalizing and empathy into more homogeneous units. The central result from this analysis was a separation of brain activation patterns into three clusters—cognitive, affective, and intermediate—which we will describe in the following sections. For ease of reference in the context of the present review article, we refer to the three clusters from our previous meta-analysis by “cognitive/mentalizing”, “affective/empathy”, and “intermediate mentalizing/empathy”.

**Figure 1:**
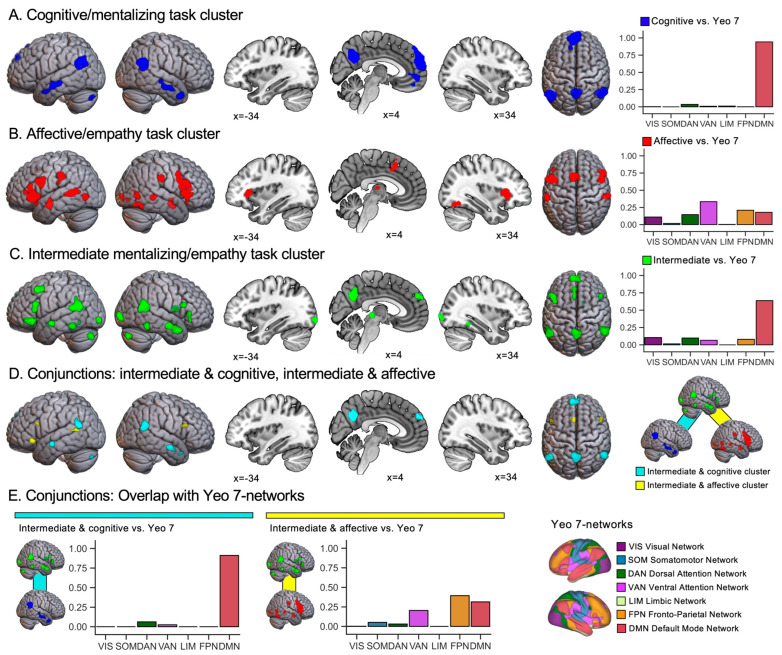
(**A**–**C**) Results of the meta-analytic clustering of brain activation for mentalizing and empathy by Schurz et al. (2021) after re-analysis with the activation likelihood estimation (ALE) method. The accompanying bar charts indicate the proportion of activation for each rendered map that falls into each resting-state network of the Yeo 7-network atlas (Yeo et al., 2011). Maps show the results for (**A**) cognitive task cluster largely reflecting abstract mentalizing, (**B**) affective task cluster reflecting concrete and sensory-based empathy, and (**C**) intermediate task cluster reflecting combined mentalizing and empathy. (**D**) Results from conjunction analyses of cognitive and intermediate (turquoise) and affective and intermediate (yellow) maps. (**E**) Proportion of activation from the conjunction maps falling into each resting-state network of the Yeo 7-network atlas. A cluster-forming threshold of *p* < 0.001 and a cluster-level threshold of *p* < 0.05 (family-wise error corrected) were applied to all meta-analytic maps.

The cognitive/mentalizing cluster was found to reflect mentalizing, which requires self-generated cognition decoupled from the physical world, and comprised tasks asking participants to process stories about false beliefs, make personality trait judgments, or play strategic games that involve guessing the other player’s intentions. As shown in Figure 1A, brain activation for the cognitive/mentalizing task cluster showed activation typical for the default mode network of the brain, prominently featuring the medial prefrontal cortex, bilateral temporoparietal cortices, and precuneus.

The affective/empathy cluster captured more affective processes, which are engaged when we witness emotions in others, and are based on shared emotional, motor, and somatosensory representations. It contained tasks asking participants to merely observe or share the pain or emotions of others, as well as making complex mental state judgments based on face stimuli (reading mind in the eyes task). In terms of brain activation, the affective/empathy cluster (Figure 1B) contained attentional, premotor, and somatosensory association areas. The brain networks associated with these activations featured, to the largest extent, the ventral attention network, and in addition, the frontoparietal network, dorsal attention network, visual network, and default mode network.

The intermediate mentalizing/empathy cluster reflected combined processes, which engage the just-described cognitive and affective processes in parallel. It comprised social animation tasks (animated geometric shapes depicting social interactions), tasks asking participants to reason about intentions related to emotions or actions, and tasks asking participants to evaluate emotions in different situational contexts. As shown in Figure 1C, brain activation for the intermediate mentalizing/empathy cluster was characterized by a combination of cognitive and affective areas, such as the bilateral temporoparietal area, precuneus, medial prefrontal cortex, and left middle and inferior frontal gyri (including a sub-peak in the left insula). The brain networks associated with these activations contained, to the largest extent, the default mode network, and to lesser degrees also the visual network, dorsal and ventral attention networks, and the frontoparietal network. Taken together, these results offer a data-driven sorting of processes underlying mentalizing and empathy tasks, which improves homogeneity within and differentiation between associated brain systems.

### 1.2. Social Interaction Engagement

In the present quantitative review, we employ the described clustering to obtain a clearer picture of the overlaps between different neural components of mentalizing and empathy tasks on the one hand, and social interaction engagement on the other. Few meta-analytic comparisons exist for this question. While multiple meta-analyses can be found for the topics mentalizing (e.g., Defendini & Jenkins, 2023; Schurz et al., 2014; Van Overwalle, 2009), empathy (e.g., Ding et al., 2020; Kogler et al., 2020; Timmers et al., 2018), the intersection of mentalizing and empathy (e.g., Arioli et al., 2021; Maliske et al., 2023), and social interaction engagement alone (e.g., Cutler & Campbell-Meiklejohn, 2019; Feng et al., 2021; Gabay et al., 2014; Li et al., 2024; Rhoads et al., 2021), systematic comparisons between the three processes are rare. Arioli and Canessa (2019) carried out an extensive meta-analysis comparing the neural correlates of mentalizing, action observation, and social interaction observation. This analysis found that observing other individuals who are interacting co-recruits typical mentalizing areas (e.g., bilateral temporoparietal junction, medial prefrontal cortex) and parts of the action observation network (e.g., left posterior superior temporal sulcus, right middle occipital gyrus). In the present review, we take a different perspective on social interactions by focusing on being actively engaged in them.

It has been argued that active participation in social interaction is fundamentally different from passive observation of social stimuli (Schilbach et al., 2013), and reflects an ecologically more valid measure of social cognition associated with a complex pattern of brain activity (Redcay & Schilbach, 2019; Schilbach et al., 2013). In Schurz et al. (2021, p. 314/315), based on a provisionary review of the literature, we speculated that co-activation of cognitive and affective areas of the brain (i.e., related to both the cognitive/mentalizing and affective/empathy cluster), may be a central characteristic of such ecologically more valid forms of social cognition and, in particular, social interaction engagement.

We utilize the results from a large-scale meta-analysis of the neural correlates of social interaction engagement (Feng et al., 2021) to compare them with brain activity for mentalizing and empathy. In that meta-analysis, social interactions were defined as processes where two or more individuals are in meaningful contact, leading to changes in their behaviors (see also Merrill, 1965). Accordingly, the tasks included in the meta-analysis required participants to actively engage in reciprocal relationships, where each individual could influence the other’s behaviors and mental states.

**Figure 2:**
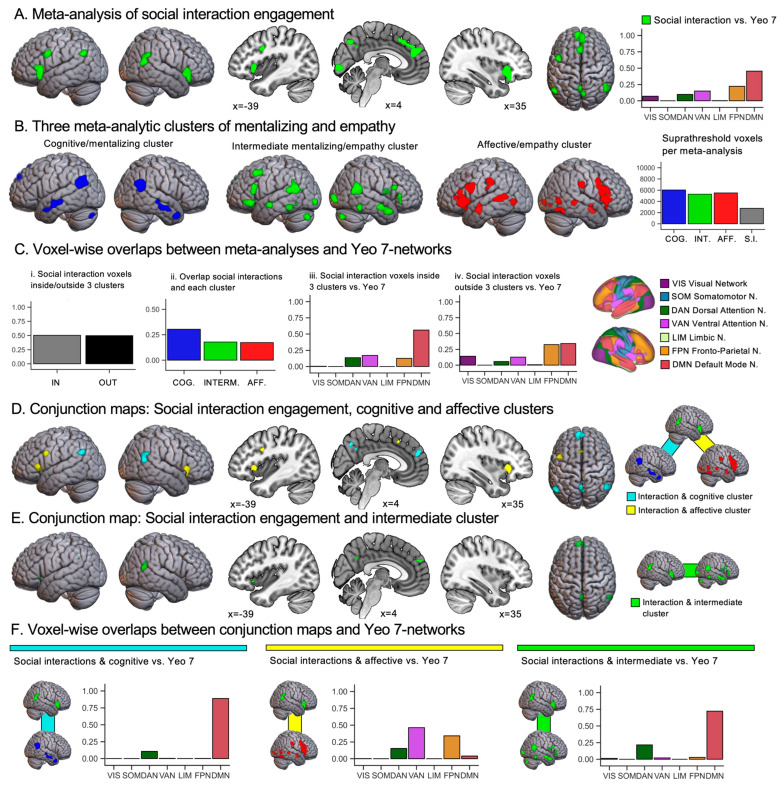
(**A**) Results of the contrast social interactions vs. nonsocial control conditions from the meta-analysis on engagement in social interactions by Feng et al. (2021), shown with a cluster-forming threshold of *p* < 0.001 and a cluster-level threshold of *p* < 0.05 (family-wise error corrected). The accompanying bar chart indicates the proportion of activation from this map that falls into each resting-state network of the Yeo 7-network atlas (Yeo et al., 2011). (**B**) For illustrative purposes, the meta-analytic result maps for the three clusters of mentalizing and empathy are shown again (cf. Figure 1) using the same statistical threshold as in (**A**). The adjacent bar chart shows the total number of suprathreshold voxels per meta-analysis featured in (**A**,**B**). (**C**) Bar charts show: (**i**) Proportion of voxels activated for the social interaction engagement meta-analysis that overlap with one or more maps of the meta-analytic clustering of mentalizing and empathy. (**ii**) Proportion of voxels of the social interaction engagement meta-analysis that overlaps with each individual meta-analytic cluster map, calculated separately per cluster map. (**iii**) Brain networks associated with regions where social interaction engagement overlapped with any of the three clusters of mentalizing and empathy. (iv) Brain networks associated with regions where social interaction engagement did not overlap with any of the three clusters. (**D**) Results of conjunction analyses showing the intersection between social interaction engagement and the cognitive/mentalizing cluster (turquoise) and affective/empathy cluster (yellow), respectively. (**E**) Results from a conjunction analysis showing an overlap between social interaction engagement and the intermediate mentalizing/empathy cluster. (**F**) Bar charts showing for each conjunction map (**D**,**E**) the proportions of activation from this map that fall within each resting-state network of the Yeo 7-network atlas.

With respect to the comparison to mentalizing and empathy, a particularly relevant aspect of Feng et al.’s (2021) meta-analysis is that the authors identified common and key areas inherently engaged by social interactions by pooling across various tasks, without prior assumptions of specialized elements. To illustrate, the relevant meta-analytic contrast social interactions > nonsocial control conditions by Feng et al. (2021) pooled activation across a multitude of structured interactive games, such as the prisoner’s dilemma game, ultimatum game, aggression tasks, trust game, cyberball game, donation tasks, social comparison tasks, dating tasks, and social conformity tasks. Furthermore, tasks in which participants were explicitly asked to empathize or mentalize about others were excluded from the analysis. As shown in Figure 2A, the authors found overarching brain activation across all tasks in areas of the default mode network (including the right temporoparietal cortex, precuneus, and medial prefrontal cortex), the ventral attention network (including the bilateral insulae), the frontoparietal/central executive network, and subcortical regions. This pattern of brain activation shows potential overlap with the results of our meta-analytic clustering of mentalizing and empathy tasks (Schurz et al., 2021). In the present quantitative review, we aim to systematically assess these overlaps, and characterize the distinct patterns of overlap for the cognitive/mentalizing, affective/empathy, and intermediate mentalizing/empathy cluster.

### 1.3. Aims of the Review

Based on the background laid out in the last sections, our review has two main aims: First, we seek to determine whether the co-recruitment of cognitive/mentalizing- and affective/empathy-like processes in general is a characteristic of social interaction engagement. Second, we explore whether there is a specifically high overlap between brain activation for the intermediate mentalizing/empathy cluster identified in our meta-analysis (Schurz et al., 2021) and activation for engagement in social interactions. As an additional aim, we examine whether overlaps between social interaction engagement, mentalizing and empathy are sensitive to the valence of interactions. This exploration is based on the results of an additional analysis carried out by Feng et al. (2021), that is, a meta-analytic comparison of the neural correlates of engaging in positive vs. negative social interactions. Finally, we examine the large-scale functional brain networks that underlie brain activation for the socio-cognitive processes featured in our review. We compare brain activation maps to resting-state networks, as the latter offer a task-free demarcation of distinct domains of brain function (Cole et al., 2016; Smith et al., 2009; Tavor et al., 2016). This, in turn, enables us to delineate different processes implicated in social cognition tasks and social interaction engagement based on a shared and theory-neutral reference.

## 2. Materials and Methods

### 2.1. Data Sources

The first source for our comparison was our previous meta-analysis (Schurz et al., 2021), from which we generated brain activation maps for the cognitive/mentalizing cluster, the affective/empathy cluster, and the intermediate mentalizing/empathy cluster. The cognitive/mentalizing task cluster summarized 57 studies featuring false belief tasks, personality trait judgments, and strategic game tasks. The affective/empathy task cluster contained 73 studies and tasks asking to passively observe pain, passively observe emotions, actively share emotions or pain, or perform complex mental state judgments based on face expressions (the reading the mind in the eyes task). The intermediate mentalizing/empathy task cluster featured 58 studies, and included the task types social animations (geometric shapes portraying social interactions), reasoning about intentions linked to emotions, reasoning about action intentions, and evaluating emotions in situational contexts.

The second source for our comparison was the meta-analysis on social interaction engagement by Feng et al. (2021). More specifically, we adopted the brain activation map from the contrast social interactions vs. nonsocial control conditions (Feng et al., 2021, Figure 2 on p. 294) for evaluating the main questions of this review. To identify common and key brain areas for social interaction engagement, activation in this map was pooled across 64 studies featuring multiple different interactive games, such as the prisoner’s dilemma game, ultimatum game, aggression tasks, trust game, cyberball game, donation tasks, social comparison tasks, dating tasks, and social conformity tasks. For our additional exploratory analysis assessing the valence-relatedness of overlaps between social interaction engagement, mentalizing and empathy, we used the maps from the contrast negative vs. positive interactions (Feng et al., 2021, Figure 4 on p. 295, 100 studies) and positive vs. negative interactions (Feng et al., 2021, Figure 3 on p. 294, 85 studies).

Before proceeding to the next analytic steps, we screened our data sources for duplicate studies. We identified eight studies overlapping between our meta-analysis on mentalizing and empathy (Schurz et al., 2021) and the social interaction meta-analysis by Feng et al. (2021). All of these studies were part of the task group strategic games and the cognitive/mentalizing cluster in Schurz et al. (2021). For the re-analysis of our meta-analysis with GingerALE software, we removed these eight studies (Assaf et al., 2009; Chaminade et al., 2012; Fukui et al., 2006; Gallagher et al., 2002; Krach et al., 2009; Riedl et al., 2014; Rilling et al., 2004; Suzuki et al., 2011; Zhou et al., 2014). Therefore, these studies are now only featured in the meta-analytic maps on social interaction engagement, and no longer in the clusters on mentalizing and empathy.

### 2.2. Meta-Analytic Procedure

To ensure comparability of meta-analytic results, we re-implemented our meta-analysis using the same methodology as Feng et al. (2021): We analyzed the input data of our meta-analysis with GingerALE 3.0.2 (https://brainmap.org/ale/ (accessed on 24 November 2024)), implementing an activation likelihood meta-analysis (ALE, Eickhoff et al., 2012, 2017; Turkeltaub et al., 2002), adopted a cluster-level family-wise error (cFWE) corrected *p* < 0.05 with a cluster-forming threshold of *p* < 0.001, and carried out 10,000 permutations.

### 2.3. Voxel-Wise Overlap Analysis

To determine voxel-wise overlaps between maps, we used the SPM12 image calculator (www.fil.ion.ucl.ac.uk/spm (accessed on 25 February 2025)). We employed a modified version of the Dice score to measure the extent of overlap. For a given meta-analysis map, we assessed the proportion of voxels falling within each comparison map (i.e., another meta-analytic map or connectivity network map). Therefore, with i1 being our meta-analysis map and i2 being a comparison map, we calculated (n voxels in i1&i2)/(n voxels in i1). By calculating proportions scaled to the total number of voxels for the given meta-analysis map, we sought to maintain a consistent metric despite variations in the overall size across the maps (e.g., maps for individual meta-analyses vs. maps from the conjunction of meta-analyses). For labelling the activation clusters in our maps, we used the “cluster” function of the command-line utilities in FSL 5.0 (https://fsl.fmrib.ox.ac.uk/fsl/ (accessed on 25 February 2025)). Image overlaps are displayed with MRIcroGL (Rorden & Brett, 2000).

### 2.4. Network-Level Mapping

For mapping meta-analysis maps in terms of large-scale networks of the brain, we employed the widely used Yeo 7-network atlas (Yeo et al., 2011), specifically the MNI-space version “liberal mask” (https://surfer.nmr.mgh.harvard.edu/fswiki/CorticalParcellation_Yeo2011 (accessed on 21 March 2025)). Before quantifying overlaps, we adjusted all meta-analysis maps in terms of size and resolution to Yeo et al.’s (2011) atlas, i.e., we carried out an SPM reslice job using the Yeo 7-network map as image defining space. Furthermore, as the Yeo 7-network atlas covers only cortical but not subcortical areas, we restricted (masked) all of our meta-analysis maps to these cortical areas before calculating the overlaps.

## 3. Results

Figure 1A–C show the results of our meta-analytic clustering of mentalizing and empathy tasks (Schurz et al., 2021), after repeating the meta-analyses with GingerALE (see Supplementary Table S1 for details). For a brain-network-based perspective, bar charts in Figure 1A–C show the proportion of overlap for each meta-analytic map and the seven networks identified by Yeo et al. (2011). The cognitive task cluster, associated with abstract mentalizing, exhibited brain activation predominantly in the default mode network. The largest activation clusters were observed in the medial prefrontal cortex, bilateral temporoparietal cortices, and precuneus. In contrast, the affective task cluster, linked to concrete and sensory-based empathy, showed activation distributed across attentional, premotor and somatosensory association areas. The largest activation clusters were identified in the bilateral insulae, inferior frontal and precentral gyri, supplementary motor area, and bilateral supramarginal and middle temporal gyri. These activations implicated the ventral attention, frontoparietal, dorsal attention, visual, and default mode networks. The activation pattern of the intermediate task cluster, which reflects a combination of mentalizing and empathy (Figure 1C), incorporated elements from both the cognitive and affective clusters. The largest clusters of activation were observed in the bilateral temporoparietal area, precuneus, medial prefrontal cortex, and left inferior and middle frontal gyri. The brain networks associated with these activations encompassed the default mode (most widely implicated), visual, dorsal attention, ventral attention and frontoparietal networks. Figure 1D further specifies the brain regions activated for the intermediate cluster, which overlap with cognitive and affective cluster areas. Cognitive regions overlapping with the intermediate cluster (1782 voxels in total) were primarily situated within the default mode network (Figure 1E) and included, for example, the precuneus, medial prefrontal cortex, and bilateral temporoparietal area. Affective regions (394 voxels in total) included the bilateral inferior frontal, left middle temporal, and superior frontal gyri. These affective components were observed across frontoparietal, default mode, and ventral attention networks.

Figure 2A shows the results of the meta-analysis on engagement in social interactions (Feng et al., 2021; see Supplementary Table S1 for details). The largest clusters of activation were found in the medial prefrontal cortex, bilateral temporoparietal cortices, bilateral insulae, and precuneus. As shown in the bar chart in Figure 2A, the activations for social interaction engagement implicated mostly the default mode, ventral attention, and frontoparietal networks. Figure 2B illustrates again the three clusters from our meta-analysis of mentalizing and empathy, and additionally reports the number of suprathreshold voxels per meta-analysis map in a bar chart. In Figure 2C, we quantify the overlaps between the meta-analysis on social interaction engagement and the meta-analytic clustering of mentalizing and empathy. As shown in Figure 2C(i), we found that approximately half of the suprathreshold voxels for the social interaction engagement meta-analysis overlapped with one or more maps of the meta-analytic clustering of mentalizing and empathy. As shown in Figure 2C(ii), the largest overlap was found with the map of the cognitive/mentalizing cluster (842 voxels in total). The affective/empathy and intermediate mentalizing/empathy clusters exhibited a comparable degree of overlap (479 and 477 voxels, respectively), which was less extensive than that of the cognitive/mentalizing cluster. Next, we individually mapped brain network profiles for regions engaged in social interaction, separating those that overlapped with mentalizing or empathy clusters from those that did not. Figure 2C(iii) shows that the regions exhibiting overlap were mainly linked to the default mode network and, to a lesser extent, to the ventral attention, dorsal attention, and frontoparietal networks. Regions without such overlap, as shown in Figure 2C(iv), were mainly associated with the default mode and frontoparietal networks, and to a lesser extent with visual, ventral attention, and dorsal attention networks.

In Figure 2D,E and Table 1, we report the overlaps between meta-analyses. Figure 2D shows overlaps between the social interaction engagement, cognitive/mentalizing cluster and affective/empathy cluster maps. We found overlaps between social interaction engagement and the cognitive/mentalizing cluster in in the bilateral temporoparietal area (angular and posterior middle temporal gyri), medial prefrontal cortex and bilateral precuneus (see Figure 2D). As shown in Figure 2F, these activations were almost entirely located in the brain’s default mode network, and to a small extent also in the dorsal attention network. Overlaps between social interaction engagement and the affective/empathy cluster were found in the bilateral insulae, left inferior frontal gyrus, and left superior frontal gyrus. As shown in a bar chart in Figure 2F, these overlaps were mainly located in the ventral attention, frontoparietal, and dorsal attention networks. In Figure 2E, we show overlaps between social interaction engagement and the intermediate mentalizing/empathy cluster. These overlaps were located in the medial prefrontal cortex, right posterior middle temporal gyrus (i.e., temporoparietal cortex), and left precuneus. Additionally, a small cluster of overlap was found in the left insula. In terms of brain networks, reported in Figure 2F, activations were mainly located in the default mode network and, to a minor extent, also in the dorsal attention network.

Table 1 further reports the results from our exploratory analysis of the role of valence. For all overlaps found between social interaction engagement and the cognitive/mentalizing, affective/empathy, and intermediate mentalizing/empathy cluster, we report whether a given cluster overlaps with the brain activation maps for the contrasts negative > positive or positive > negative interactions computed by Feng et al. (2021), which we indicate by “N > P” or “P > N” in the leftmost column of Table 1. No overlaps (negative > positive, positive > negative) were found for the cognitive/mentalizing cluster. For the intermediate mentalizing/empathy cluster, an overlap was only found for the small cluster of activation in the insula. For this brain region, activation was higher for negative > positive social interactions. No overlaps were found for the reverse contrast of valence. For the affective/empathy cluster, we found large areas of overlap in bilateral insulae (>100 voxels) and smaller overlap in the left superior frontal gyrus (50 voxels). These overlaps imply that the key areas of overlap between social interaction engagement and the affective/empathy cluster also show a valence effect for social interactions, i.e., higher activation in the negative > positive social interaction contrast.

## 4. Discussion

In this review, we investigated whether the co-recruitment of brain areas implicated in mentalizing and empathy is a characteristic of social interaction engagement. To address this question, we compared the results of two previously published meta-analyses. The first meta-analysis identified task clusters of mentalizing and empathy based on their associated brain activation patterns (Schurz et al., 2021). The second meta-analysis summarized brain activation associated with social interaction engagement (Feng et al., 2021). We hypothesized that the co-activation of systems implicated in processing cognitive and affective mental states represents a central characteristic of social interaction engagement.

### 4.1. Social Interaction Engagement Co-Recruits Cognitive and Affective Processes

Overall, the results of our review support the assumption that social interaction engagement co-recruits cognitive and affective brain systems that are also implicated in mentalizing and empathy. However, we did not find evidence for a specifically high overlap with the intermediate mentalizing/empathy cluster.

In more detail, approximately 50% of the suprathreshold voxels in the social interaction engagement map intersected with one or more of the mentalizing/empathy clusters (Figure 2C(i)). While the three meta-analytic brain activation maps from clustering mentalizing and empathy were roughly comparable in size (approximately 5000–6000 voxels), the map for social interaction engagement was about half as large (~2800 voxels). With respect to the overlaps we found, two questions are central to our discussion.

Our first question was whether social interaction engagement would show a specifically high overlap with the intermediate mentalizing/empathy cluster identified in our meta-analysis (Schurz et al., 2021). The results shown in Figure 2C(ii) provide little support for this hypothesis. We found the largest overlap with social interaction engagement for the cognitive/mentalizing cluster. The intermediate mentalizing/empathy and affective/empathy clusters showed comparable levels of overlap, which were considerably lower than that found for the cognitive/mentalizing cluster (~30% vs. ~18% for cognitive/mentalizing vs. other maps).

The second question addressed in our review was whether the co-recruitment of cognitive/mentalizing- and affective/empathy-like processes in general reflects a characteristic of ecologically more valid social cognition tasks, such as engaging in social interactions. The results shown in Figure 2D support this assumption. We found overlaps between social interaction engagement and the cognitive/mentalizing cluster in the medial prefrontal cortex, bilateral temporoparietal cortices (angular gyri), and precuneus. These overlaps include areas commonly considered core regions for mentalizing (see Molenberghs et al., 2016; Schurz et al., 2014). Moreover, we found overlaps between social interactions and the affective/empathy cluster including areas considered central for empathy (Fan et al., 2011; Timmers et al., 2018), that is, the bilateral insulae, and a small cluster in the superior frontal gyrus (with the peak 3 mm dorsal to the mid-cingulate cortex).

### 4.2. Effects of Valence on Social Interaction Engagement

Our main hypotheses were focused on the key neural processes engaged by social interaction engagement, determined by pooling across various tasks without prior assumptions of specialized elements. After determining how these processes overlap with mentalizing and empathy, we carried out an additional exploratory analysis to assess whether the identified overlaps were also sensitive to the valence of interactions. This analysis was based on an additional contrast computed in the meta-analysis by Feng et al. (2021), comparing positive vs. negative social interactions across a range of different tasks, such as exclusion vs. inclusion in the cyberball game, win vs. loss in competition tasks, or fairness vs. unfairness in the ultimatum game. Prominent valence-related effects were only found for overlaps between social interaction engagement and the affective/empathy cluster. Corresponding valence-related effects for the cognitive/mentalizing and the intermediate mentalizing/empathy cluster were absent or rather small (<20 voxels). Overlaps between social interaction engagement and the affective/empathy cluster showed higher activation for negative vs. positive social interactions in bilateral insulae and the supplementary motor area (see Table 1). We note that negative feelings are not only integral for the contrast of negative versus positive social interactions in Feng et al.’s (2021) meta-analysis, but also a common feature of the tasks in the affective/empathy cluster. The majority of the latter tasks featured negative emotion or pain as the target state, specifically the task groups observing pain, observing emotions, and sharing emotions or pain. Another interesting parallel regards common activation in the insula for self- and other-related processing. For empathy, research shows common activation in the insula for first-hand experience and vicarious experience of pain (e.g., Carrillo et al., 2019; Corradi-Dell’Acqua et al., 2016; Lamm et al., 2011; Rütgen et al., 2015); these and similar findings gave rise to “shared network” accounts of empathy (e.g., Gallese & Goldman, 1998; Preston & de Waal, 2002). Also, for social interaction engagement, a meta-analysis (Yang et al., 2019) found activation in bilateral insulae both when participants themselves were non-cooperative, or when participants were confronted with a non-cooperative player (i.e., participants enacted/experienced violations of cooperation, unfairness, or betrayal). Yang et al. (2019) argued that insula activation, sensitive to cooperation violation both by self and others, is related to aversive feelings which might be essential for motivating cooperative behavior. This notion complements earlier research highlighting the importance of motivational processes for collaborative as opposed to competitive interactions (Decety et al., 2004; Le Bouc & Pessiglione, 2013).

### 4.3. A Brain Network Perspective

Our results suggest that co-activation of cognitive and affective brain regions is an overarching characteristic of the intermediate mentalizing/empathy cluster and social interaction engagement (see Figure 1D and Figure 2D, respectively). Moreover, for both social interactions and the intermediate cluster, we observed higher overlap with cognitive compared to affective regions. In addition, as bar charts in Figure 1C and Figure 2A illustrate, both social interactions and the intermediate cluster co-localized most highly with the default mode network. However, social interactions revealed additional overlaps with the so-called control networks of the brain (e.g., Cole et al., 2014; Dosenbach et al., 2008)—frontoparietal, ventral attention, and dorsal attention networks. These overlaps were less prominent, but not entirely absent, for the intermediate mentalizing/empathy task cluster: In total, 24% of that cluster’s activation was located within the three control networks (see Figure 1C).

Notably, the pattern of default mode and control network co-activation was also observable for the affective/empathy cluster and was more prominent than that for the intermediate cluster of mentalizing/empathy tasks. In general, the default mode network and control networks frequently exhibit competitive and inhibitory patterns of interaction. Research indicates that during passive rest, the default mode and ventral attention networks are functionally unrelated (e.g., Alcalá-López et al., 2018) or even display activity that is negatively correlated (Bzdok et al., 2013; Chai et al., 2012; Fox et al., 2005; Zhou et al., 2018). When tasks require placing attention on external stimuli, the activity of the default mode network is reduced (e.g., Mazoyer et al., 2001; Raichle et al., 2001; Shulman et al., 1997). Studies have demonstrated that the ventral attention network can inhibit the default mode network to minimize interference from non-task-related processes (Goulden et al., 2014; Wen et al., 2013; Trautwein et al., 2016).

On the other hand, for tasks that demand high cognitive effort, large-scale networks have been found to enhance their integration, that is, to form positive associations. For example, studies found increases in global network integration for higher levels of n-back working memory tasks (e.g., Shine et al., 2016; Vatansever et al., 2015; Wendelken et al., 2016). Shine et al. (2016) further demonstrated that network integration increases during multiple cognitive tasks compared to passive rest, with a mentalizing task (social animations) and an n-back working memory task showing the highest integration. Identifying both segregation and integration within networks does not imply a contradiction; rather, it reflects two opposing constraints on cognitive function (Shine & Poldrack, 2018). Segregation promotes functional specialization, whereas integration enhances neural flexibility through more controlled and effortful processing (Kitzbichler et al., 2011; Shine & Poldrack, 2018; Vatansever et al., 2015). Therefore, social interaction engagement and social cognition tasks with higher complexity and ecological validity found among the intermediate and affective clusters of mentalizing and empathy might be characterized by the functional integration of brain networks which otherwise operate alone (see also Schurz et al., 2020).

### 4.4. Limitations and Future Directions

The patterns of overlap we found suggest that the processes engaged by social interaction engagement and the three clusters of mentalizing and empathy are functionally related but not identical. One factor that may explain this limited correspondence is that the tasks used to probe mentalizing, empathy, and intermediate mentalizing/empathy differ from those used to study social interaction engagement. The meta-analysis of mentalizing and empathy (Schurz et al., 2021) focused on the processing of cognitive and affective mental states of other people. Therefore, participants’ own beliefs, feelings, and motivation were not directly subject to judgments. In contrast, the meta-analysis on engagement in social interactions (Feng et al., 2021) featured a range of tasks where participants spontaneously considered the intentions (and possibly emotions) of other people when making their own decisions. When making such decisions for oneself, affective areas arguably also process one’s own valuation of the task (e.g., the rewards or punishments obtained currently). This highlights (part of) the additional complexity of social interaction engagement: Besides the mental states of others, one’s own affective state and motivation are crucial for decision-making in social interactions.

Another limitation of our review is that we compared thresholded meta-analytic maps. Although this may offer a useful overview for comparing findings across related topics, the approach is statistically limited. While we ensured a coherent methodological approach and the application of the same statistical threshold across meta-analyses, a joint meta-analysis based on coordinate data files of individual studies (~the “raw data” of meta-analyses) would offer more flexibility and statistical power for examining convergence and divergence between social interactions, mentalizing and empathy.

A third limitation of our review is that our brain network-based perspective is limited to cortical large-scale resting state networks included in the Yeo 7-network atlas (Yeo et al., 2011), leaving subcortical regions outside the scope. In this regard, we note that the results from the social interaction engagement meta-analysis featured a cluster of activation with its peak in the right insula, further extending to subcortical regions outside the Yeo 7-network atlas, specifically the right putamen and caudate nucleus. These subcortical activations showed only marginal overlap with the meta-analysis maps of mentalizing and empathy. However, subcortical areas like the putamen and caudate nucleus are relevant for social interaction engagement, possibly reflecting motivational and reward-related processes (Haber & Knutson, 2010; Yang et al., 2019).

Taken together, while the meta-analytic comparison presented in this review synthesizes across a large number of studies to identify the most common overlaps between social interaction engagement, mentalizing and empathy, it does not replace insights generated by empirical approaches. To further our understanding of the overlaps between social interaction engagement, mentalizing, and empathy, analyses of task-based fMRI using functional connectivity and graph-theoretical techniques, as well as multimodal approaches relating brain activation measurements to other modalities, such as ecological momentary assessments, could provide fruitful avenues for future research.

## 5. Conclusions

The observations of our review highlight that social interaction engagement co-recruits cognitive and affective brain systems implicated in mentalizing and empathy. We found limited evidence for a specific overlap between brain activation for social interaction engagement and a cluster of intermediate mentalizing/empathy tasks found in our previous meta-analysis (Schurz et al., 2021). Instead, the largest overlaps were observed with the cognitive/mentalizing cluster. We further found that social interaction engagement, affective/empathy and intermediate mentalizing/empathy tasks involve a common pattern of large-scale network engagement. Specifically, we found co-activation of the default mode network and control networks, including the ventral attention, dorsal attention, and frontoparietal networks. These results indicate that integrated processing of networks that by default are functionally segregated (e.g., Chai et al., 2012; Shulman et al., 1997) might be an overarching characteristic of social interaction engagement and social cognition tasks with higher complexity and ecological validity featured in affective/empathy and intermediate mentalizing/empathy tasks.

## Figures and Tables

**Table 1:** Overlaps between social interaction engagement meta-analysis and meta-analytic clustering of mentalizing and empathy.

	Cluster Peak					Sub-Peaks			
AAL/Yeo 7-Networks/Neg. vs. Pos.	x	y	z	Z-Val.	vx	x	y	z	AAL/Yeo 7-Networks
*Overlap: Social interaction engagement and Cognitive/mentalizing tasks*									
L sup. front. g./DMN/-	−8	52	34	5.14	330	4	50	26	R ant. cing. g./DMN
						−6	54	18	L sup. front. g./DMN
R mid. temp. g./DAN/-	56	−56	16	5.09	232	50	−50	28	R angular g./DMN
L angular g./DMN/-	−52	−62	34	4.29	145				
Precuneus/DMN/-	0	−54	34	4.57	99				
R precuneus/DMN/-	6	−64	38	3.76	36				
*Overlap: Social interaction engagement and Intermediate mentalizing/empathy tasks*									
L sup. front. g./DMN/-	−6	54	34	5.13	188	4	50	30	R sup. front. g./DMN
						8	48	26	R ant. cing. g./DMN
R mid. temp. g./DAN/-	56	−56	16	5.09	172	52	−52	28	R angular g./DMN
Precuneus/DMN/-	0	−54	34	4.57	99				
L insula/VAN/N>P 18 vx	−38	20	−6	3.67	18				
*Overlap: Social interaction engagement and Affective/empathy tasks*									
R insula/FPN/N>P 178 vx	34	22	0	4.94	180	40	22	−12	R inf. front. g./DMN
L insula/VAN/N>P 119 vx	−32	24	6	4.72	135	−40	18	0	L insula/FPN
L inf. front. g./DAN/-	−46	6	30	4.75	115				
L sup. front. g./FPN/N>P 50 vx	−2	18	44	3.94	50				

*Note.* Results were obtained by voxel-wise conjunction, Z-values report the minimum statistic across maps, and results are reported at a minimum cluster size of 10 voxels. We compared the social interaction engagement meta-analysis by Feng et al. (2021) contrasting social interactions vs. nonsocial control conditions with the meta-analytic clusters of mentalizing and empathy reported by Schurz et al. (2021). DAN … dorsal attention network, VAN … ventral attention network, FPN … frontoparietal network, DMN … default mode network, Neg. vs. Pos. … N vs. P … results from the contrasts negative vs. positive social interactions, vx … voxel.

## Data Availability

No new data were created in this review. Coordinate files and result maps for the re-analysis of Schurz et al. (2021) are available upon request.

## Supplementary Materials

The following supporting information can be downloaded at: https://www.mdpi.com/article/10.3390/bs15070934/s1, Table S1: Results of the Social Interaction Engagement Meta-Analysis (Feng et al., 2021) and the Re-Analysis of the Meta-Analytic Clusters of Mentalizing and Empathy (Schurz et al., 2021).
