# Supplementary material for: A Quantitative Review of Brain Activation Maps for Mentalizing, Empathy, and Social Interactions: Specifying Commonalities and Differences
Source: Behav Sci (Basel). 2025 Jul 10;15(7):934. doi: 10.3390/bs15070934 (PMC12293034; doi:10.3390/bs15070934)
Supplement: Supplementary file 1 [file behavsci-15-00934-s001.zip › behavsci-3590188-supplementary.pdf]

Supplementary Table S1

Results of the Social Interaction Engagement Meta-Analysis (Feng et al., 2021) and the Re-Analysis of the Meta-Analytic Clusters of Mentalizing and Empathy (Schurz et al., 2021).

	Cluster Peak					Sub-Peaks			
AAL/Yeo 7-Networks	x	y	z	Z-/ALE-Val.	vx	x	y	z	AAL/Yeo 7-Networks
Engaging in social interactions meta-analysis (Feng et al., 2021)									
L. sup. front. g. / VAN	-2	22	42	5.22	768	-6	52	34	L sup. front. g. / DMN
						6	48	26	R ant. cing. g. / DMN
R insula / FPN	32	22	-6	6.59	500	50	20	-16	R sup. temp. pole / LIM
						12	22	-4	R caudate / -
L insula / VAN	-34	18	-2	6.29	453	-32	20	-6	L insula / FPN
R mid. temp. g. / DAN	54	-60	14	5.39	334	50	-50	30	R angular g. / DMN
L precentral g. / DAN	-44	4	32	6.19	187	-40	16	30	L inf. front. g. / FPN
L calcarine sulcus / VIS	2	-86	-2	4.93	163				
L angular g. / DMN	-52	-62	34	4.29	156				
R precuneus / DMN	8	-66	40	4.41	108				
L precuneus / DMN	0	-54	34	4.57	99				
Cognitive/mentalizing cluster of mentalizing and empathy meta-analysis (Schurz et al., 2021)									
R sup. front. g. / DMN	4	58	18	0.0524	2368	-4	48	46	L sup. front. g. / DMN
						0	52	-6	L med. orb. front. g. / DMN
						0	44	-18	L g. rectus / LIM
L angular g. / DMN	-50	-58	26	0.0688	889				
L precuneus / DMN	2	-56	30	0.0742	840				
R angular g. / DMN	56	-54	26	0.0705	806				
L mid. temp. g. / DMN	-60	-10	-14	0.0347	452	-62	-22	-10	L mid. temp. g. / DMN
R mid. temp. g. / DMN	56	-18	-16	0.0259	382	60	-6	-18	R mid. temp. g. / DMN
R temp. pole / DMN	50	12	-32	0.0331	191				
L cerebellum, crus II / -	-26	-78	-36	0.0218	112				
Intermediate mentalizing/empathy cluster of mentalizing and empathy meta-analysis (Schurz et al., 2021)									
R mid. temp. g. / DMN	54	-48	16	0.0478	678				
L precuneus / DMN	-4	-54	36	0.0421	635	0	-60	34	L precuneus / DMN
L mid. temp. g. / VAN	-54	-52	16	0.0431	611	-52	-52	32	L angular g. / DMN
						-52	-68	14	L mid. temp. g. / VIS
R mid. temp. g. / DMN	60	-8	-16	0.0443	449	50	8	-26	R mid. temp. g. / DMN
L sup. front. g. / DMN	-6	54	34	0.038	319	8	48	26	R ant. cing. g. / DMN
						6	60	28	R sup. front. g. / DMN
L inf. front. g. / DMN	-46	26	-8	0.0306	252	-38	20	-6	L insula / VAN
L mid. front. g. / DMN	-44	16	46	0.0414	249				
L inf. front. g. / DMN	-52	22	12	0.0315	240	-50	20	22	L inf. front. g. / FPN

AAL/Yeo 7-Networks	Cluster Peak x	y	z	Z-/ALE-Val.	vx	Sub-Peaks x	y	z	AAL/Yeo 7-Networks
L sup. front. g. / DMN	-4	32	52	0.0363	225	-4	18	50	L supp. motor / FPN
R inf. occ. g. / VIS	30	-94	0	0.0422	209				
L inf. temp. g. / DAN	-40	-46	-16	0.0294	165	-44	-58	-12	L inf. occ. g. / DAN
R cerebellum, crus I / -	26	-78	-34	0.0334	164				
L cerebellum, crus II / -	-22	-80	-36	0.0355	159				
R caudate / -	12	4	12	0.0404	154				
- / -	-4	-26	-2	0.0291	150	4	-26	-2	- / -
R fusiform g. / DAN	42	-46	-18	0.0314	141				
L mid. temp. g. / DMN	-60	-10	-14	0.0338	140				
L mid. occ. g. / VIS	-32	-92	-6	0.0371	122				
R inf. front. g. / DMN	56	28	4	0.0296	121	50	30	-8	R inf. front. g. / DMN
R inf. front. g. / FPN	48	18	20	0.0319	110				
Affective/empathy cluster of mentalizing and empathy meta-analysis (Schurz et al., 2021)									
R insula / VAN	30	18	6	0.0392	1294	52	22	24	R inf. front. g. / FPN
						50	6	38	R precentral g. / DAN
L inf. front. g. / DMN	-52	30	-4	0.0339	913	-42	16	0	L insula / VAN
						-38	26	-2	L inf. front. g. / DMN
L supp. motor / VAN	-6	14	48	0.051	569				
L inf. front. g. / FPN	-48	8	26	0.0353	560	-48	12	26	L inf. front. g. / FPN
						-58	10	28	L precentral g. / DAN
L mid. temp. g. / DMN	-58	-44	6	0.0348	371	-56	-52	8	L mid. temp. g. / VAN
L hippocampus / -	-16	-8	-14	0.0328	302				
L supramarginal g. / DAN	-58	-26	38	0.0462	246	-58	-22	24	L supramarginal g. / VAN
R supramarginal g. / VAN	64	-24	32	0.0324	228	52	-24	32	R supramarginal g. / DAN
R fusiform g. / VIS	40	-58	-20	0.0264	182	36	-70	-16	R fusiform g. / VIS
R amygdala / -	22	-4	-16	0.0299	180	18	-6	-6	- / -
L inf. occ. g. / DAN	-44	-68	-2	0.0368	151				
R mid. temp. g. / VIS	48	-64	0	0.0291	135	48	-74	10	R mid. temp. g. / VIS
L thalamus / -	-2	-14	6	0.0252	135	-10	-14	10	L thalamus / -
R mid. temp. g. / DMN	52	-36	-2	0.0383	129				
R calcarine sulcus / VIS	14	-92	0	0.0292	124	16	-80	-8	R lingual g. / VIS

Note. A cluster-forming threshold of $p < 0.001$ and a cluster-level threshold of $p < 0.05$ (family-wise error corrected) were applied to all meta-analytic maps. For the meta-analysis on social interaction engagement, peak voxel activations are reported as Z-values, as provided by Feng et al. (2021; see Table 1, p. 293). For the mentalizing/empathy clusters, peak voxel values are based on thresholded ALE images (i.e., the main result of GingerALE meta-analysis), and thus reflect ALE values. For the conjunction analysis presented in the main text,

however, we relied on Z-score images additionally generated by GingerALE, and thus all maps used for the conjunction analyses contained the same units. Results are reported at a minimum cluster size of 10 voxels. VIS … Visual Network, DAN … Dorsal Attention Network, VAN … Ventral Attention Network, LIM … Limbic Network, FPN … Frontoparietal Network, DMN … Default Mode Network, vx … voxel.
